# Humanin, a Mitochondrial-Derived Peptide Released by Astrocytes, Prevents Synapse Loss in Hippocampal Neurons

**DOI:** 10.3389/fnagi.2019.00123

**Published:** 2019-05-31

**Authors:** Sandra Cristina Zárate, Marianela Evelyn Traetta, Martín Gabriel Codagnone, Adriana Seilicovich, Analía Gabriela Reinés

**Affiliations:** ^1^Instituto de Investigaciones Biomédicas (INBIOMED, UBA-CONICET), Facultad de Medicina, Universidad de Buenos Aires, Buenos Aires, Argentina; ^2^Departamento de Histología, Embriología, Biología Celular y Genética, Facultad de Medicina, Universidad de Buenos Aires, Buenos Aires, Argentina; ^3^Instituto de Biología Celular y Neurociencias “Prof. E. De Robertis” (IBCN, UBA-CONICET), Facultad de Medicina, Universidad de Buenos Aires, Buenos Aires, Argentina; ^4^Departamento de Farmacología, Facultad de Farmacia y Bioquímica, Universidad de Buenos Aires, Buenos Aires, Argentina

**Keywords:** astrocytes, ovarian hormones, mitochondria, humanin, synapse, hippocampus

## Abstract

Astroglial cells are crucial for central nervous system (CNS) homeostasis. They undergo complex morpho-functional changes during aging and in response to hormonal milieu. Ovarian hormones positively affect different astroglia parameters, including regulation of cell morphology and release of neurotrophic and neuroprotective factors. Thus, ovarian hormone loss during menopause has profound impact in astroglial pathophysilogy and has been widely associated to the process of brain aging. Humanin (HN) is a secreted mitochondrial-encoded peptide with neuroprotective effects. It is localized in several tissues with high metabolic rate and its expression decreases with age. In the brain, humanin has been found in glial cells in physiological conditions. We previously reported that surgical menopause induces hippocampal mitochondrial dysfunction that mimics an aging phenotype. However, the effect of ovarian hormone deprivation on humanin expression in this area has not been studied. Also, whether astrocytes express and release humanin and the regulation of such processes by ovarian hormones remain elusive. Although humanin has also proven to be beneficial in ameliorating cognitive impairment induced by different insults, its putative actions on structural synaptic plasticity have not been fully addressed. In a model of surgical menopause in rats, we studied hippocampal humanin expression and localization by real-time quantitative polymerase chain reaction (RT-qPCR) and double immunohistochemistry, respectively. Humanin production and release and ovarian hormone regulation of such processes were studied in cultured astrocytes by flow cytometry and ELISA, respectively. Humanin effects on glutamate-induced structural synaptic alterations were determined in primary cultures of hippocampal neurons by immunocytochemistry. Humanin expression was lower in the hippocampus of ovariectomized rats and its immunoreactivity colocalized with astroglial markers. Chronic ovariectomy also promoted the presence of less complex astrocytes in this area. Ovarian hormones increased humanin intracellular content and release by cultured astrocytes. Humanin prevented glutamate-induced dendritic atrophy and reduction in puncta number and total puncta area for pre-synaptic marker synaptophysin in cultured hippocampal neurons. In conclusion, astroglial functional and morphological alterations induced by chronic ovariectomy resemble an aging phenotype and could affect astroglial support to neuronal function by altering synaptic connectivity and functionality. Reduced astroglial-derived humanin may represent an underlying mechanism for synaptic dysfunction and cognitive decline after menopause.

## Introduction

Proper functioning of the central nervous system (CNS) requires the tight intercommunication between two main cell types: neurons and glial cells. It is now well accepted that glia functions go much beyond mere structural and metabolic support to neurons, indeed they are now recognized as key players in diverse physiological processes such as synaptic communication and plasticity, homeostasis and network-level activity in the adult brain (Allen and Lyons, 2018). Neuron-glia interplay is highly dynamic and prone to changes as a result of the natural process of aging as well as alterations associated to different pathologies (Verkhratsky et al., 2014). During aging, both structural and physiological changes that occur in the brain have been attributed to changes in glial cells, which reduce their number and modify gene expression (Palmer and Ousman, 2018). In fact, glial rather than neuronal-specific genes have been proposed to be better predictors of age (Soreq et al., 2017). Among glial cells, astrocytes are crucial for maintaining synaptic connectivity throughout life by means of creating a perisynaptic sheath that accumulates molecules responsible for synaptic support (Verkhratsky et al., 2015). Aged astrocytes undergo profound morpho-functional alterations, which involve retraction of their cytoplasmic processes and a decline in the production of metabolic and trophic factors. These alterations have a direct impact in CNS health, as aged astrocytes reduce their neuroprotective and homeostatic capacity, altering, in turn, neuronal synaptic connectivity and functionality (Verkhratsky et al., 2010, 2014; Palmer and Ousman, 2018).

Ovarian hormones estradiol and progesterone have trophic effects involved in the maintenance of both reproductive and non-reproductive functions in different tissues (Nilsen and Brinton, 2002; Morrison et al., 2006). In the brain, they exert potent antioxidant and neuroprotective actions that promote cognitive health. In fact, their loss during aging and natural or induced menopause has been linked to several pathological conditions, such as neuroinflammation, mitochondrial dysfunction, synaptic decline, cognitive impairment and increased risk of neurodegenerative disorders (Zárate et al., 2017b). It has been reported that the density of dendritic spines as well as synapse number in the CA1 area of the hippocampus decrease with natural or surgical loss of ovarian hormones (Gould et al., 1990; Woolley and McEwen, 1992, 1993; Adams et al., 2001). Many of the beneficial effects of these hormones are mediated through their direct actions on neurons. However, astrocytes are also cellular targets of ovarian hormones and thus are highly involved in the protective and reparative actions of estradiol and progesterone (Acaz-Fonseca et al., 2014). Through binding to sex hormone receptors in astrocytes, ovarian hormones regulate several cellular, molecular and functional parameters in these cells, including the growth of astroglial cytoplasmic processes, the expression of glial fibrillary acidic protein (GFAP), glutamate transport and the release of neurotrophic and neuroprotective factors (Acaz-Fonseca et al., 2014, 2016; Palmer and Ousman, 2018). Considering the active role of astrocytes in regulating synaptic maintenance and plasticity and the dependence on ovarian hormone signaling for several of their neuroprotective functions, it is expected that decline in sex hormones after menopause results in impaired astroglial synaptic function.

Humanin (HN) is a cytoprotective 24 amino acid peptide which was originally isolated from a cDNA library constructed from nervous tissue of a patient with familial Alzheimer’s disease (AD). Since then, it has been identified in different species such as mice, nematodes and rats (Niikura et al., 2004). The rat humanin homolog rattin (HNr) is a 38 amino acid peptide encoded and translated from an open reading frame (ORF) within the mitochondrial 16S ribosomal RNA (rRNA) gene (Caricasole et al., 2002; Paharkova et al., 2015). Several studies have demonstrated that HN is a potent pro-survival factor for neurons exposed to multiple cell stressors, such as Aβ oligomers and over-expression of familial AD-related genes (Hashimoto et al., 2001; Caricasole et al., 2002), serum deprivation (Kariya et al., 2002), stroke (Xu et al., 2006; Gao et al., 2017) and *N*-methyl-D-aspartate (NMDA)-induced excitotoxicity (Cui et al., 2014). HN and its derivatives have also proven to be beneficial in ameliorating cognitive impairment induced by Aβ, muscarinic receptor antagonists and aging in rodents (Mamiya and Ukai, 2001; Krejcova et al., 2004; Tajima et al., 2005; Niikura et al., 2011; Zhang et al., 2012; Yen et al., 2018). Moreover, HN and HNr were reported to prevent Aβ-induced spatial learning and memory impairments in rats by a mechanism involving changes in long-term potentiation (Chai et al., 2014; Wang et al., 2014), synaptic protein expression, dendritic branch number and spine density in the hippocampus (Chai et al., 2014).

It has been reported that HN exerts its neuroprotective action from the extracellular space through binding to a trimeric IL-6-receptor-related receptor(s) on the cell surface involving the receptor for ciliary neurotrophic factor α (CNTFR-α), WSX-1 and glycoprotein 130 kDa (gp130) subunits (Matsuoka and Hashimoto, 2010) and further modulation of tyrosin kinase, ERK1/2, AkT, STAT3 and JNK signaling cascades (Hashimoto et al., 2005; Matsuoka and Hashimoto, 2010; Takeshita et al., 2013; Kim et al., 2016). HN has been ubiquitously detected in different adult tissues with high metabolic rate, including skeletal and cardiac muscle, cerebral cortex, hippocampus and liver both in humans and in rodents (Caricasole et al., 2002; Kariya et al., 2005; Muzumdar et al., 2009). The mechanisms regulating HN expression are not fully elucidated. It has been shown that HN levels decrease with age in human and mice plasma as well as in the rat hypothalamus (Muzumdar et al., 2009; Bachar et al., 2010). Also, there is a sexual dimorphism in the expression of HN in rat anterior pituitary cells, suggesting that sex hormones are involved in the regulation of HN biosynthesis in this gland (Gottardo et al., 2014). In the brain, HN has been widely localized both in neurons and glial cells, albeit the former only in pathological conditions (Tajima et al., 2002). Thus, glial cells have been suggested to be the main production sites of this peptide in the brain in physiological conditions (Niikura et al., 2004). However, astroglial HN expression and release and the regulation of such processes by ovarian hormones have not been studied so far.

All things considered, the aim of this work was to evaluate the expression of HNr in the hippocampus of a rat model of surgical-induced menopause. We also aimed at determining whether astroglia is able to produce and release HNr *in vitro* and ovarian hormone dependence on these processes. HN putative actions on structural synaptic plasticity in a model of glutamate-induced dendritic atrophy were also studied.

## Materials and Methods

### Drugs

All drugs and reagents were obtained from Sigma Chemical Co., St. Louis, MO, USA except for Dulbecco’s Modified Eagle Medium (DMEM) and supplements (Gibco, Invitrogen Carlsbad, CA, USA) fetal calf serum (FCS; Natocor, Córdoba, Argentina) and the materials indicated below.

### Animals

Adult female Wistar rats were housed in groups of four in controlled conditions of light (12 h light-dark cycles) and temperature (20–22°C). Rats were fed standard lab chow and water *ad libitum* and kept in accordance with the National Institutes of Health Guide for the Care and Use of Laboratory Animals. Animal protocols were previously approved by the Ethics Committee of the School of Medicine, University of Buenos Aires (Res. No. 2249).

For *in vivo* experiments, rats were ovariectomized (OVX) or sham-operated (SHAM) at 3 months of age under ketamine (100 mg/kg, i.p.) and xylazine (10 mg/kg, i.p.) anesthesia and ketoprofen (5 mg/kg) for analgesia. Beginning on week 10 post-surgery, rat hormonal status was monitored daily by vaginal smears. SHAM animals had 4–5 days estrous cycles while OVX animals presented continuous diestrus status. Eleven weeks after the surgery, rats were subjected to behavioral tests as described below. Twelve weeks after the surgery, rats were either deeply anesthetized (100 mg/kg ketamine and 6 mg/kg xylazine, i.p.), transcardially perfused with heparinized saline solution and fixed with 4% paraformaldehyde in 0.1 M phosphate buffer (for free-floating immunostaining of brain sections) or euthanized in a CO_2_ chamber followed by decapitation (for real-time quantitative polymerase chain reaction (RT-qPCR) assays).

### Behavioral Tests

#### Open Field Test

The open field test was performed to evaluate animal general locomotor activity and exploratory behavior (Gould et al., 2009). The arena consisted of a squared open field (60 × 60 cm) limited by a 40 cm-height wall with a grided floor divided into squares (15 × 15 cm) by lines. Animals were individually placed in the center of the open field arena and were allowed to freely explore for 10 min. The frequency with which the animal crossed grid lines with all four paws (crossings) was recorded as a measure of locomotor activity. After each animal was tested, the open field was cleaned with a 10% ethanol-damp cloth. Testing was performed between 10:00 and 14:00 h in a quiet room illuminated with a 75 W electric bulb, hung 75 cm above the open field apparatus.

#### Y-Maze Spontaneous Alternation Test

Spontaneous alternation behavior in a Y-maze was recorded to evaluate animal spatial working memory (Miedel et al., 2017). The apparatus consisted of three identical black arms (50 × 10 × 40 cm, length × width × height). Animals were habituated to the testing room for at least 30 min prior to the test. At the beginning of the session, animals were placed individually at the end of one same arm of the Y-maze and allowed to freely explore for 6 min. The whole session was recorded using a SONY CCD-TRV75 video camera recorder connected to a personal computer with AVerTV A833 video capture. The number of total arm entries and the number of triads (referred as entries to a different arm of the maze in each of three consecutive arm entries) were recorded and the percentage of alternation was calculated as [number of alternations/(total arm entries-2)] × 100 (Miedel et al., 2017).

#### Elevated Plus Maze

The elevated plus maze test was carried out to evaluate animal anxiety-like behavior (Walf and Frye, 2007). The apparatus consisted of a plus-shaped maze containing two open arms (50 × 10 cm, length × width) and two enclosed arms by 40 cm high walls arranged such that the two open arms were opposite to each other. The apparatus was placed on four legs so that it was elevated 50 cm off the floor. Testing was performed between 10:00 and 16:00 h in a quiet room illuminated with a dim light hung 75 cm above the center of the maze. At the beginning of the session, each animal was placed in the center of the maze facing a close arm and allowed to freely explore for 5 min. The whole session was recorded and behavior was assessed offline. The number of times the animal entered an arm with all four paws was recorded. The percentage of open arm entries was calculated as [number of open arm entries/total arm entries] × 100.

#### Forced Swimming Test

The forced swimming test was performed to evaluate animal depressive-like behaviors by quantifying their mobility and immobility and associated behaviors (Overstreet, 2012). Each rat was individually placed in a plastic cylinder (diameter, 40 cm; height, 35 cm) containing water (23−25°C) up to 25 cm from the bottom for 5 min. At 5 s intervals throughout the test session, the predominant behavior was assigned to one of the followings categories: (1) immobility: lack of movement, except for the ones needed to keep the head above water; (2) swimming: swimming movement throughout the cylinder or (3) climbing: vigorous movements of the forepaws in and out of the water, usually directed against the walls. After each animal was tested, the water was changed and the cylinder rinsed with clean water. Following the session, each animal was dried and placed to its housing cage in a temperature-controlled room. All swimming sessions were carried out between 10:00 and 16:00 h.

### RNA Isolation and Reverse-Transcription Real-Time Quantitative Polymerase Chain Reaction (RT-qPCR)

Immediately after decapitation, hippocampi from SHAM and OVX rats were dissected on ice, snap-frozen and kept at −80°C until use. Total RNA was extracted from frozen tissue using QuickZol reagent (Kalium Technologies, Buenos Aires, Argentina) according to the manufacturer’s protocol. One μg of total RNA was treated with 2 U DNAse (Promega Corp., Madison, WI, USA) and then reverse transcribed using SuperScript II Reverse Transcriptase (Invitrogen, Thermo Fisher Scientific) following the manufacturer’s instructions. Amplification of the products from RT reactions was performed in duplicate using specific primers (HNr forward 5′-GAG GGT TCA ACT GTC TCT TAC TTT CA-3′, reverse 5′-GTG AAG AGG CTG GAA TCT CCC-3′; HPRT forward 5′-CTC ATG GAC TGA TTA TGG ACA GGA C-3′, reverse 5′-GCA GGT CAG CAA AGA ACT TAT AGC C-3; Invitrogen, Thermo Fisher Scientific) and SYBR Green Select Master Mix (Invitrogen, Thermo Fisher Scientific) on a StepOne™ Real-Time PCR System (Applied Biosystems, Thermo Fisher Scientific). PCR product specificity was verified by a melting curve analysis. Negative RT controls were performed by omitting the addition of the reverse transcriptase enzyme in the RT reaction, while negative template controls were performed by addition of nuclease-free water instead of cDNA. Both primer sets were pre-validated to check similar efficiency ~2 and for the use of 2^−ΔΔCt^ method as the quantification method (Livak and Schmittgen, 2001). Lack of statistically significant variation of endogenous reference gene HPRT expression between SHAM and OVX animals was determined (Supplementary Figure S1). Gene expression was normalized to HPRT using Step-One Software (Applied Biosystems, Thermo Fisher Scientific), and expressed as fold-changes relative to the control group.

### Free-Floating Immunostaining of Tissue Sections

After animal intracardiac perfusion, brains were dissected, postfixed with 4% paraformaldehyde in 0.1 M phosphate buffer and equilibrated in 25% (w/v) sucrose in the same buffer. The hippocampus was serially sectioned in a freezing microtome and the free-floating coronal 30-μm-thick tissue sections were stored at −20°C in 25% (w/v) sucrose in phosphate buffer until use. The sections were blocked with 10% (v/v) normal goat serum and incubated for 48 h with primary antibodies against HNr (1:1,500), anti-GFAP (1:500, Millipore), NF-200 (1:1,000), OLIG2 (1:50,000, Millipore) or S100B (1:1,000) followed by an hour incubation with Alexa 594- or FITC-labeled secondary antibodies (Jackson ImmunoResearch). 4′,6 diamidino-2-phenilindoledihydrocloride (DAPI) was used for DNA staining. Negative controls were incubated in the absence of primary antibodies (Supplementary Figure S2).

### Astroglial Cell Culture

Astroglial cell cultures were prepared from neonatal rat pups of 3–4 days old. Hippocampi and cortices from four to six pups were isolated, cut into small fragments using scissors and then cells were mechanically dispersed by extrusion through a Pasteur pipette in Hank’s Balanced Salt Solution. After decanting tissue, supernatant was transferred to a new tube and pelleted. The procedure was repeated twice and finally the cells were plated in poly-D-lysine coated bottles with high glucose DMEM supplemented with 10% FCS, 100 μg/ml penicillin-streptomicin (DMEM-S) and 1 μg/ml fungizone. After the first 24 h and every 3–4 days, media was replaced with fresh DMEM-S. When the cells reached confluence (10–12 days), they were subjected to shaking at 180 rpm for two consecutive 24 h-periods at 37°C to detach microglia and oligodendrocytes. The cells were then incubated with 0.625% 5-fluorouracil for further 24 h, washed and incubated in fresh DMEM supplemented with 10% FCS previously treated with 0.025% dextran-0.25% charcoal (FCS-DCC) to remove steroids for additional 48 h. Then the cells were tripsinized and re-seeded onto 12 well plates in DMEM FCS-DCC for 24–48 h. Cultures obtained with this procedure showed 93 ± 0.04% GFAP-positive astrocytes (*n* = 3 independent cultures), as reported in the literature (Villarreal et al., 2014). Then, the cells were cultured for 4 h in DMEM FCS-DCC containing 1 nM 17β-estradiol (E) and 1 μM progesterone (P) or vehicle (ethanol, 20 μl/l), washed and cultured for further 20 h in fresh DMEM FCS-DCC.

### Expression of HNr in Astroglial Cells by Flow Cytometry

Cultured astroglial cells were harvested with 0.025% trypsin-EDTA, washed in cold PBS, fixed with 4% paraformaldehyde and permeabilized with 0.1% saponin in PBS (MP Biomedicals Inc., OH, USA) for 10 min. Then, the cells were incubated with rabbit anti-HNr antibody (1 μg/μl) in PBS-0.05% saponin for 1 h at 37°C followed by 40-min incubation with a FITC-conjugated anti-rabbit secondary antibody (1:100) in the same buffer. To determine the cut-off for HNr fluorescence, cells were incubated with secondary antibody only. Cells were washed, resuspended in PBS and analyzed by flow cytometry using a FACScan (Becton Dickinson). Data were analyzed with WinMDI 98 software.

### ELISA

HNr levels in supernatants from astroglial cultures were measured by ELISA using a commercial kit HN(N) (Rat)-EIA Kit (Phoenix Pharmaceuticals) following the manufacturer’s instructions and normalized to 50 μg of total protein in cell lysates from corresponding wells. Attached astrocytes were harvested as described above. Cells were lysed in lysis buffer containing 150 mM NaCl, 1% Igepal, 0.02% sodium azide, 0.1% sodium dodecyl sulfate (SDS), in 50 mM Tris-HCl pH 7.4 and a protease inhibitor cocktail (1:50). Cell lysate was collected and clarified by centrifugation at 16,000× *g* for 30 min and total protein content was determined by the Bradford protein assay (BioRad Laboratories, CA, USA) using bovine serum albumin as standard.

### Metabolic Activity of Viable Cells

The metabolic activity of viable cells was determined by the 3-(4,5-dimethylthiazol-2-yl)-2,5-diphenyltetrazolium bromide (MTT) assay (Promega, Madison, WI, USA). Cells were washed twice and incubated for 4 h in 100 μl Krebs buffer plus 50 μg MTT reagent in PBS at 37°C. The developed crystals were dissolved in 100 μl 0.04 N HCl in isopropanol and the OD was read in a microplate spectrophotometer at a wavelength of 600 nm. The quantity of formazan product is directly proportional to the number of living cells in culture.

### Primary Neuronal Cultures and Glutamate Treatment

Hippocampal neuronal cultures were prepared from dissected embryonic day 18 hippocampi as previously reported (Reinés et al., 2012; Podestá et al., 2014). Briefly, hippocampal tissue was trypsinized and mechanically dissociated. Then, dispersed cells were seeded on poly-D-lysine-coated glass coverslips at a density of 2 × 10^4^ cells/cm^2^ in Neurobasal medium supplemented with 2% (v/v) B27 and 0.5 mM glutamine. On day 13 *in vitro* (DIV), neurons were treated with 5 μM glutamate or vehicle for 3 min at 37°C, media was immediately removed and cells were washed with Hank’s balanced salt solution. Immediately after, neurons were incubated with Humanin peptide (HN, 0.01–1 μM; Genemed Synthesis, Inc., San Antonio, TX, USA) in supplemented culture media for 24 h and then fixed for immunostaining as described below.

### Immunostaining of Fixed Cells

Neurons in culture (14 DIV) were fixed in 4% (w/v) paraformaldehyde/4% (w/v) sucrose in PBS solution, pH 7.2 for 20 min at room temperature (RT) and permeabilized with 0.2% (v/v) Triton X-100 for 10 min at RT followed by a blockade with 5% (v/v) normal goat serum for 1 h at RT. The cells were then incubated overnight at 4°C with MAP-2 (1:500) or SYN (1:2,000; Chemicon, Millopre) primary antibodies in PBS. The next day, cells were washed and incubated for 1 h at RT with the appropriate secondary antibodies followed by DAPI for DNA staining. Negative controls were incubated in the absence of primary antibodies (Supplementary Figure S3). Finally, cells were mounted using Mowiol.

### Image Acquisition and Quantification

A Zeiss Axiophot microscope (Carl Zeiss, Oberkochen, Germany) equipped with an Olympus Q-Color 5 camera or a Olympus IX81 microscope equipped with a CCD model DP71 digital camera were employed in immunohistochemistry or immunocytochemistry assays, respectively. Confocal images were acquired using Fluoview version 3.3 software using an Olympus FV300 confocal microscope. Immuno-positive structures were quantified as relative immunoreactive area (immunoreactive area/total area) as described elsewhere using the ImageJ (NIH) software (Reinés et al., 2008; Aviles-Reyes et al., 2010; Codagnone et al., 2015). In immunohistochemistry assays, the total area corresponded to the hilus, granular and molecular layers in the superior and inferior blades of the dentate gyrus (DG) and to pyramidal and stratum lucidum and radiatum in CA1. Briefly, microscopic images were captured with a digital camera, transformed to an 8-bit gray scale and an interactive threshold was determined. Then, the area fraction covered by immunostained structures obtained using this threshold, which remained fixed for the entire experiment, was quantified with the particle counting tool of the software. In immunocytochemistry assays, dendritic tree area per neuron was calculated by subtracting the area corresponding to the immunolabeled soma from the total MAP-2 immunostaining. The number of synaptic puncta, total puncta area and individual puncta area for SYN was calculated as previously described (Podestá et al., 2014). Figures were prepared using Adobe Photoshop 7.0 software (Adobe Systems Inc.). Brightness and contrast were kept constant between the experimental groups. Each immunohistochemistry assay consisted of 5–6 hippocampal serial sections of each animal per group. The average data obtained from the quantification of sections from the same animal was considered *n* = 1. Results of immunohistochemistry assays are expressed as mean values (±SEM) of *n* = 3–5 animals per group. Each experiment was repeated 2–4 times. Each immunocytochemistry assay consisted of 1–3 coverslips per experimental condition. Results of immunocytochemistry assays are expressed as mean values (±SEM) of 20–40 neurons per experimental condition from two to three independent cultures. The experiments were repeated at least twice.

### Statistical Analysis

Results are expressed as mean ± SEM and evaluated by unpaired Student’s *t*-test or nonparametric Mann-Whitney *U* test. HNr content in conditioned media determined by ELISA was evaluated by one-way ANOVA followed by Tukey’s test. Dendritic tree area, total SYN puncta area, number and individual SYN puncta area were evaluated by two-way ANOVA followed by Tukey’s test. Differences were considered significant if *p* < 0.05.

## Results

### Behavioral Characterization of OVX-Induced Model of Menopause

**Figure 1:**
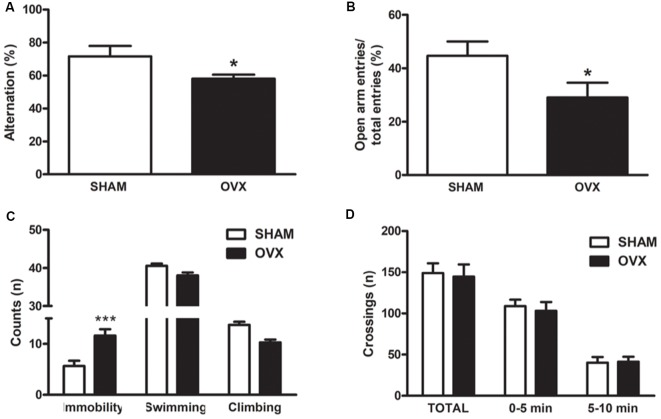
Behavioral characterization of an animal model of surgical menopause in adult rats. Adult Wistar female rats were ovariectomized (OVX) or sham-operated (SHAM). Twelve weeks after surgery, the animals were subjected to behavioral tests as described in “Materials and Methods” section. **(A)** Y-maze spontaneous alternation test: for each animal, the number of total arm entries and the number of triads in 6 min was recorded. **(B)** Elevated plus maze test: each animal was allowed to freely explore for 5 min and the number of times the animal entered an arm with all four paws was recorded. **(C)** Forced swimming test: every 5 s, a time-sampling technique was used to score the presence of immobility, swimming or climbing behavior. **(D)** SHAM or OVX rats were placed individually in the center of a field marked with a grid of 16 equal squares for 10 min. The number of times the animal crossed each line was registered. Each column represents the mean ± SEM of **(A)** the percentage of alternation, **(B)** the percentage of open arm entries, **(C)** the number of counts or **(D)** the number of crossings per session (*n* = 4–12 animals/group); *p < 0.05, ***p < 0.001, Student’s *t*-test.

It is now accepted that ovariectomized (OVX) adult rodents share common features with aged animals regarding brain function, including changes in mitochondrial function, synaptic plasticity, behavior and cognition (Zárate et al., 2017b). To characterize our animal model of surgical menopause in adult Wistar rats at the behavioral level, we assessed parameters of spatial working memory, anxiety and depression, which are known to be modulated by ovarian hormones (Diz-Chaves et al., 2012; Kiss et al., 2012; Cao et al., 2013; Rodríguez-Landa et al., 2017; Hampson, 2018; da Silva Moreira et al., 2016). Twelve-week ovarian hormone-deprived rats showed lower spontaneous alternation behavior in the Y-maze, indicating impaired spatial working memory (Figure 1A). They also spent less time in the open arms and more time immobile when subjected to the elevated plus maze and the forced swimming test, respectively. These results show that long-term ovarian hormone deprivation increases anxiety-like and depressive-like behaviors (Figures 1B,C). There were no differences in spontaneous locomotion and exploratory behavior between OVX and control groups, as assessed in the open field test (Figure 1D), indicating that the observed behaviors in OVX rats cannot be attributed to altered locomotion.

### HNr Expression in the Hippocampus

**Figure 2:**
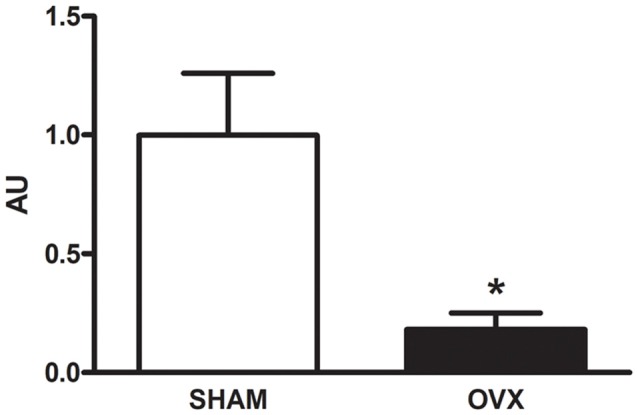
Expression of HNr in the hippocampus. The expression of HNr RNA was evaluated in hippocampi from SHAM and OVX rats by real-time quantitative polymerase chain reaction (RT-qPCR). Each column represents the mean ± SEM of the concentration of HNr RNA relative to its internal control HPRT expressed as fold-changes relative to SHAM group (AU; *n* = 3 animals/group); *p < 0.05, Student’s *t*-test.

**Figure 3:**
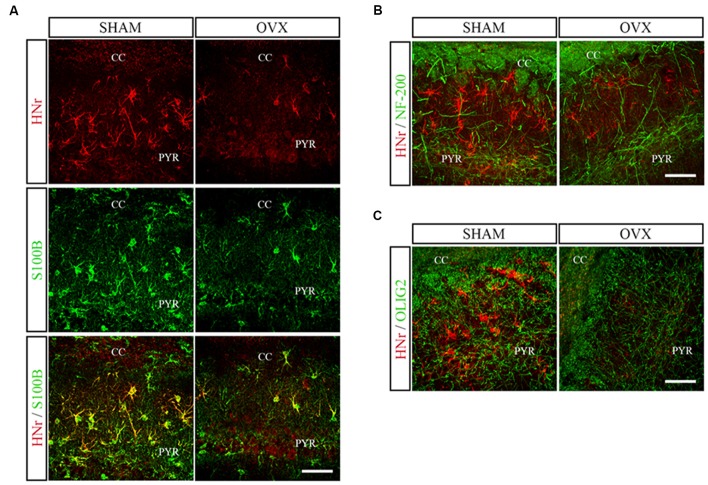
Double immunostaining for HNr and astroglial, neuronal or oligodendroglial markers. Coronal sections from the hippocampus of SHAM and OVX animals were processed for double immunohistochemistry for HNr and **(A)** astroglial (S100B), **(B)** neuronal (NF-200) or **(C)** oligodendroglial (OLIG-2) markers. Representative confocal microphotographs from CA1 stratum oriens (600×) show the expression of HNr (red), S100B, NF-200 or OLIG2 (green) and the merged image (yellow). PYR, pyramidal layer; CC, corpus callosum. Scale bar = 50 μm.

**Figure 4:**
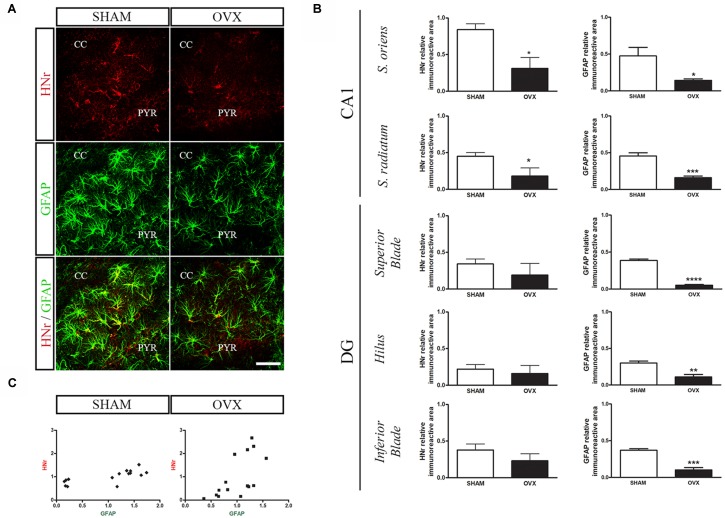
Effects of long-term ovarian hormone deprivation on HNr and glial fibrillary acidic protein (GFAP) immunoreactivity in the hippocampus. Coronal sections from the hippocampus of SHAM and OVX animals were processed for double immunohistochemistry for HNr and GFAP. **(A)** Representative confocal microphotographs from CA1 stratum oriens (600×) show the expression of HNr (red), GFAP (green) and the merged image (yellow). **(B)** Quantification of relative immunoreactive area (immunoreactive area/total area) for HNr and GFAP using ImageJ software. Each column represents the mean ± SEM of relative immunoreactive area (*n* = 3 animals/group). *p < 0.05, **p < 0.01, ***p < 0.001, Student’s *t*-test. **(C)** HNr and GFAP expression levels expressed as relative immunopositive area for each protein were normalized with respect to each corresponding hippocampal subregion (SHAM *r* = 0.74, *p* < 0.01; OVX *r* = 0.63, *p* < 0.05, Pearson correlation test). PYR, pyramidal layer; CC, corpus callosum; DG, dentate gyrus. Scale bar = 50 μm.

Circulating levels of mitochondrial-encoded HN are known to decline with age in mice and humans and in the rat hypothalamus (Muzumdar et al., 2009; Bachar et al., 2010). Indeed, this peptide has been suggested to participate in the endocrine regulation of the aging process (Lee et al., 2014). We have previously reported that long-term ovarian hormone deprivation induces functional and structural alterations in hippocampal mitochondria, which resembles a mitochondrial aging phenotype (Zárate et al., 2017a). We thus evaluated HNr expression in the hippocampus of OVX rats by RT-qPCR. Hippocampal expression of HNr was lower in OVX rats compared to control rats (Figure 2). In order to identify cell types expressing this peptide, we performed immunohistochemistry for both HNr and different brain cell markers. HNr immunostaining colocalized with astroglial marker S100B and GFAP, but not with neuronal or oligodendroglial markers NF-200 and OLIG-2, respectively (Figures 3, 4A). Noteworthy, some HNr staining could be observed in the extracellular space, suggesting the presence of secreted HNr. Further evaluation of HNr and GFAP expression levels in different hippocampal subregions showed that HNr relative immunoreactive area was lower in the CA1 region of the hippocampus of OVX rats while there was no difference between HNr relative immunoreactive area from OVX and control rats in the DG (Figure 4B). OVX rats also showed lower relative immunoreactive area for GFAP in all hippocampal subregions studied (Figure 4B). Remarkably, there was a positive correlation for HNr and GFAP levels in both OVX and control groups (Figure 4C).

### HNr Production and Release by Astroglia

**Figure 5:**
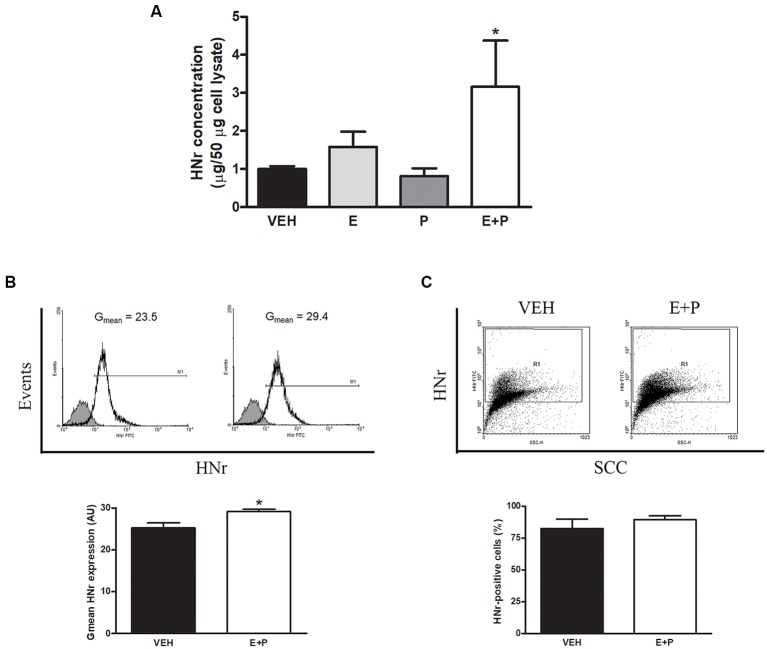
HNr production and release by astrocytes *in vitro*. Cultured astrocytes were incubated with estradiol (E) and progesterone (P). HNr secreted levels were determined in conditioned media by ELISA. Harvested cells from additional cultures were immunostained for HNr and analyzed by flow cytometry. Each column represents the mean ± SEM of **(A)** the concentration of HNr in conditioned media normalized to 50 μg of total protein in corresponding cell lysate, **(B)** the fluorescence intensity of HNr staining (Gmean) or **(C)** the percentage of HNr-positive cells. The upper panels in **(B,C)** show representative histograms and dot plots of HNr expression in astrocytes incubated with VEH or E + P (*n* = 3–4 wells per group from three independent experiments). **(A)** *p < 0.05 vs. respective control without E; ANOVA followed by Tukey’s test, **(B,C)** *p < 0.05; Student’s *t*-test.

Glial cells have been proposed as the main site of HNr production in the brain in physiological conditions (Tajima et al., 2002). Indeed, our results show that HNr immunoreactivity colocalizes with astroglial markers and that OVX rats display lower expression of HNr in the hippocampus. However, the production and release of this peptide by astroglial cells as well as the regulation of these processes by ovarian hormones remain to be determined. To address this issue, cultured astrocytes were incubated with ovarian hormones 17β-estradiol (E) and progesterone (P) alone or in combination and released HNr content was determined in the conditioned media of cultured astrocytes by ELISA. There was a two-fold increase in HNr levels in the conditioned media of astrocytes incubated with E + P (Figure 5A). Then, intracellular astroglial HNr levels were determined by immunocytochemistry and flow cytometry. Ovarian hormones increased the intracellular content of HNr per cell without changing the number of astrocytes expressing this peptide (Figures 5B,C), indicating that ovarian hormones increase both the expression and the release of HNr by these cells.

### HN Effect on Structural Synaptic Plasticity in the Hippocampus

**Figure 6:**
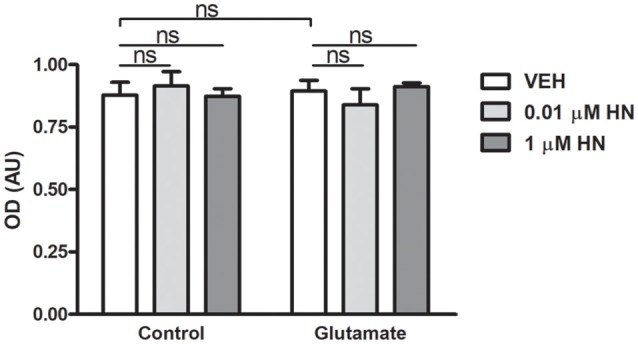
Effects of glutamate and HN on hippocampal neuronal cell viability *in vitro*. Cultured embryonic neurons (DIV13) were subjected to a 3-min incubation with glutamate (5 μM) immediately followed by a 24-h incubation with HN (0.01–1 μM). Cell viability was assessed by MTT assay. Each column represents the mean ± SEM of five wells from one experiment representative of three independent experiments. ns, non-significant; ANOVA.

**Figure 7:**
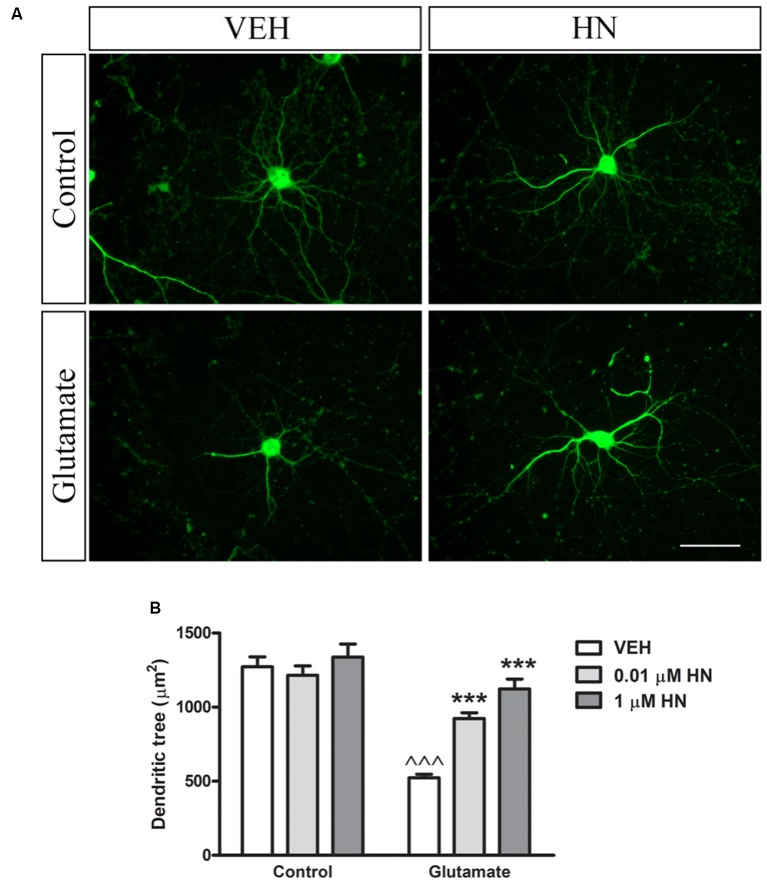
Effect of HN on neuronal dendritic tree area in hippocampal neurons *in vitro*. Hippocampal neurons in culture (DIV 13) were briefly exposed to glutamate (5 μM), immediately incubated with HN (0.01–1 μM) and evaluated 24 h later. **(A)** Representative microphotographs of hippocampal neurons in culture immunostained for MAP-2. Microphotographs corresponding to 0.01 μM HN are shown, **(B)** Quantification of MAP-2 immunostaining using ImageJ software. Each column represents the mean ± SEM of 20–40 neurons per experimental condition. ^∧∧∧^*p* < 0.01 vs. respective control without glutamate, ***p < 0.001 vs. respective control without HN; two-way ANOVA followed by Tukey’s test. Scale bar = 50 μm.

**Figure 8:**
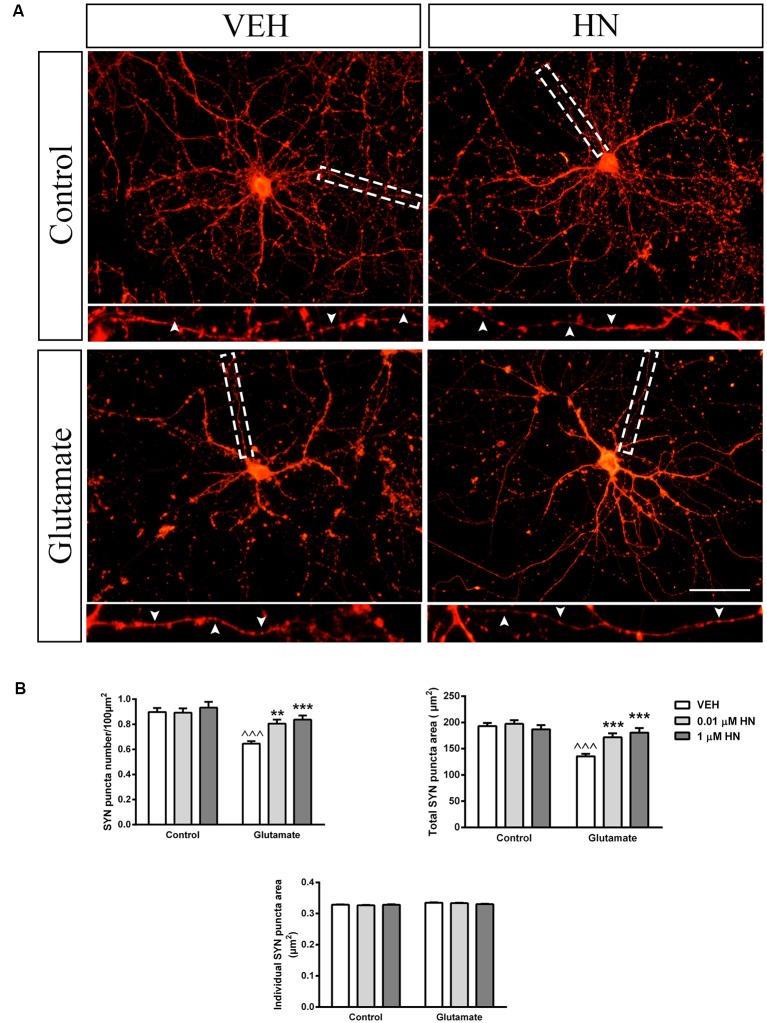
Effect of HN on SYN synaptic profile in hippocampal neurons *in vitro*. Hippocampal neurons in culture (DIV 13) were briefly exposed to glutamate (5 μM), immediately incubated with HN (0.01–1 μM) and evaluated 24 h later. **(A)** Representative microphotographs of hippocampal neurons in culture immunostained for SYN. Microphotographs corresponding to 0.01 μM HN are shown. Insets (3× magnification) detail SYN immunostaining pattern. **(B)** Quantification of total SYN puncta area, SYN puncta number and individual SYN puncta area using ImageJ software. Each column represents the mean ± SEM of 20–40 neurons per experimental condition. ^∧∧∧^*p* < 0.01 vs. respective control without glutamate, **p < 0.01, ***p < 0.001 vs. respective control without HN; two-way ANOVA followed by Tukey’s test. Scale bar = 50 μm.

It has been extensively reported that ovariectomy decreases the number of synapses and induces dendritic alterations in hippocampal neurons (Gould et al., 1990; Woolley and McEwen, 1992, 1993; Adams et al., 2001). However, HN effects on these synaptic parameters have not been explored yet. To this aim, cultured hippocampal neurons were subjected to a brief exposure to a low glutamate concentration, a condition that has previously been reported to induce dendritic atrophy in the absence of neuronal death (Podestá et al., 2014). Immediately after glutamate exposure, neurons were incubated with HN in two different concentrations and cell viability and synaptic parameters were studied 24 h later. Neither glutamate nor HN affected cell viability, as assessed by MTT assay (Figure 6). As expected, immunostaining for the specific dendritic marker MAP-2 was reduced 24 h after glutamate insult, rendering into a decreased neuronal dendritic area. Remarkably, HN prevented glutamate-induced dendritic atrophy in both concentrations studied (Figure 7). Pre-synaptic marker SYN immunostaining was also altered in glutamate-treated neurons, as previously reported (Podestá et al., 2014). Remarkably, while glutamate decreased SYN puncta number and total puncta area, both concentrations of HN prevented glutamate actions over SYN synaptic profile. Neither glutamate nor HN induced changes in SYN individual puncta area (Figure 8).

## Discussion

Mitochondrial DNA has been classically described as a maternally-inherited, small DNA encoding only 13 mitochondrial proteins involved in oxidative phosphorylation and 24 structural RNAs required for their translation. However, recent research has challenged this view (Capt et al., 2016; Luo et al., 2018). In fact, several mitochondrial-derived peptides encoded as genes-within-genes in short ORFs dispersed throughout the mitochondrial genome have been recently described (Capt et al., 2016; Kim et al., 2017). Among them, humanin (HN) has received much attention due to its neuroprotective effect against different types of stress and disease models (Lee et al., 2013). Encoded within the 16S rRNA, HN has been detected in several tissues and in circulation in rodents and humans (Caricasole et al., 2002; Kariya et al., 2005; Muzumdar et al., 2009) and its expression is age-dependent (Muzumdar et al., 2009; Bachar et al., 2010). HN circulating levels both in mice and humans are regulated by the growth hormone and insulin-like growth factor-1 (GH/IGF-1) axis, a well conserved endocrine system that controls the process of aging. In fact, HN has been suggested to be a mitochondrial signaling peptide that is secreted and acts as a hormone involved in the endocrine regulation of the aging process (Lee et al., 2014). We previously reported that 12-week ovarian hormone deprivation, a model of surgical menopause, induces mitochondrial dysfunction in the rat hippocampus (Zárate et al., 2017a). These alterations comprise slower active respiration and ATP production rates as well as decreased membrane potential together with changes in the lipid composition of mitochondrial membranes. Remarkably, these functional and structural features are found in mitochondria from aged animals (Pamplona, 2008; Gómez and Hagen, 2012), further supporting the idea that ovarian hormone loss promotes an accelerated aging phenotype, as previously suggested (Yao et al., 2009). Ovarian hormones, especially estrogens, are well-known regulators of mitochondrial function, which is crucial in organs and tissues with high energy demand like the CNS (Zárate et al., 2017b). Estrogen regulation of mitochondrial metabolism, biogenesis and morphology has been reported to occur in neuronal tissue (Garcia-Segura et al., 1998; Nilsen and Brinton, 2003; Arnold et al., 2008; Brinton, 2008; Hara et al., 2014; Kemper et al., 2014; Klinge, 2017). Since ovarian hormone loss has been associated to the process of aging and estrogens are well-known regulators of mitochondrial gene expression (Virbasius and Scarpulla, 1994; Kang et al., 2007), we hypothesized that the levels of a mitochondrial-encoded gene like HN would be decreased in hormone-deprived animals. Indeed, herein we found that long-term ovariectomy reduced the expression of HNr in the hippocampus. It is to note that, although initially transcribed as long polycistronic precursor transcripts (Ojala et al., 1981), the levels of individual mitochondrial RNAs varies in different tissues and cell types due to post-transcriptional processing mechanisms (Mercer et al., 2011; Sanchez et al., 2015). Such mechanisms have been shown to respond to the tissue hormonal milieu (Sanchez et al., 2015). In fact, estrogen treatment has been shown to increase the amount of mitochondrial-encoded RNAs (Stirone et al., 2005; Klinge, 2008). Moreover, 16S rRNA levels have been reported to decrease with aging due to reduced rate of mitochondrial RNA transcription (Calleja et al., 1993). Remarkably, female rats express more 16S rRNA than males of the same chronological age (Borras et al., 2003), suggesting a putative role of sex hormones in the regulation of its expression. Considering that HNr is encoded within the mitochondrial 16S rRNA molecule, it can be speculated that similar post-transcriptional processing mechanisms induced by hormonal environment can affect HNr expression. Also, HN peptide seems to be associated with lipids in different tissues (Tajima et al., 2002), suggesting that alterations in tissue lipid composition induced by age or disease may affect HN effective levels. Interestingly, hippocampal mitochondria membranes from OVX rats display an altered lipid profile consisting of increased membrane peroxidability index together with decreased cardiolipin levels (Zárate et al., 2017a), which could also affect HN levels and/or distribution within this tissue.

Different cells comprise the highly specialized and complex nervous tissue. Although an early report showed the presence of HN immunoreactivity in both normal and AD human brains (Tajima et al., 2002), the specific cell types expressing this peptide remained elusive. Tajima et al. (2002) showed HN immunoreactivity to be located in round cells resembling glia widely distributed in the brain, mainly in the hippocampus. However, the identity of such glial cells was not investigated then. HN immunoreactivity was also detected in some neurons of the occipital lobe in the AD brain but not in an age-matched normal brain (Tajima et al., 2002). By means of double immunohistochemistry using neuronal, astroglial and olidodendroglial markers in the hippocampus, we detected HNr immunoreactivity to be located only in astrocytes, both in OVX and control animals. However, co-immunostaining with microglial markers was not studied and HNr localization in microglial cells cannot be ruled out. Considering that microglia are highly regulated by both estrogen and progesterone, further studies are warranted to elucidate whether microglial cells also express HNr *in vivo*.

The role of astroglial cells as supportive cells in the CNS is well established. They are not only the major contributors to cell homeostasis in the CNS but also play a central role in the control of synaptic transmission through different mechanisms. Perisynaptic astroglial membranous sheaths are known to cover a high number of all synaptic contacts in the hippocampus, thus finely regulating synaptic transmission by means of establishing physical contact with synapses and also by secreting a plethora of bioactive agents (Verkhratsky and Nedergaard, 2018). These astroglial processes have been reported to be devoid of organelles but may contain mitochondria, which, in light of our results, could be the source of mitochondria-derived peptides with a local role in the neighboring synapse. Considering the effect of HN in the prevention of glutamate-induced structural synaptic alterations shown herein, this peptide could be considered as another bioactive molecule locally secreted by astroglia to regulate synaptic plasticity in pathophysiological conditions. In this way, our results add new evidence for astroglial role as a major contributor to proper signal transmission in the CNS and therefore to higher cognitive function.

*In vitro* assays using cultured astrocytes further confirmed that this cell type is able to produce and release HNr and that ovarian hormones positively regulate these processes. It is well known that estradiol and progesterone exert their actions on astrocytes through classical and non-classical receptor signaling initiated at the nucleus, membrane or cytoplasm levels (Acaz-Fonseca et al., 2016). By inducing the transcription of nuclear-encoded mitochondrial transcription factor A (TFAM), estrogens are able to promote transcription of mitochondrial DNA (mtDNA) through a classical mechanism (Virbasius and Scarpulla, 1994; Kang et al., 2007). Also, estrogen and progesterone receptors have been localized within mitochondria, suggesting that they might regulate mitochondrial transcription through direct binding to hormone-response element-like sequences in mtDNA (Demonacos et al., 1996; Chen et al., 2004). Further studies are warranted to determine ovarian hormone mechanism involved in HNr expression and release by astroglial cells.

Estradiol and progesterone are well-known regulators of astroglial cell morphology and GFAP expression. Evidence supporting this includes changes in the surface density of GFAP-immunoreactive cells in the DG of the hippocampus along the estrous cycle (Luquin et al., 1993). Moreover, GFAP-immunoreactive cell density in this brain area decreases after ovariectomy and is increased by treatment with estradiol alone or in combination with progesterone in a dose-dependent manner (Luquin et al., 1993). It has been suggested that ovarian hormones promote the increase in the size and/or branching of cell processes without affecting astrocyte cell number (Tranque et al., 1987). We evidenced the presence of qualitatively smaller, less complex astrocytes with thinner cytoplasmic processes, which resulted in lower GFAP relative immunoreactive area in all subregions of the hippocampus from OVX animals. Similar alterations in astrocyte phenotype have been described in aged rodents, primates and humans (Castiglioni et al., 1991; Amenta et al., 1998; Kanaan et al., 2010; Cerbai et al., 2012; Jyothi et al., 2015; Robillard et al., 2016). For example, Cerbai et al reported the presence of fewer, smaller and less complex GFAP-positive astrocytes in the CA1 region of the hippocampus from 22-month old rats (Cerbai et al., 2012). These morphological changes are accompanied with loss of homeostatic function, which represents an underlying mechanism for impaired neuroprotection and disrupted neuronal connectivity (Verkhratsky et al., 2014). Indeed, several recent reports have shown that the atrophic astrocyte is the main astroglial phenotype not only in natural aging but also in the early stages of neurodegenerative diseases (Verkhratsky et al., 2014). This phenotype is characterized not only by smaller and less complex astrocytes but also by a decrease in glutamate uptake and glutamate synthase activity, which may contribute to the observed general imbalance in both excitatory and inhibitory neurotransmission as well as alterations at the synapse level (Verkhratsky et al., 2014). It is of note that astrocyte cytoplasmic processes form close structural contacts with synapses to regulate all aspects of synaptic function *via* the secretion of several factors (Pfrieger, 2010). Thus, it can be speculated that OVX may negatively impact astroglial support to neuronal function by means of reduced synapse maintenance.

Accumulating evidence indicates that the CA1 region of the hippocampus highly responds to natural or surgical ovarian hormone loss by decreasing synapse number and spine density in rats (Gould et al., 1990; Woolley and McEwen, 1992, 1993; Adams et al., 2001), which has been directly associated to cognitive impairment. Interestingly, quantification of HN immunoreactivity in the hippocampus of OVX rats evidenced lower HN protein levels in the CA1 region without changes in the DG between experimental groups, which was temporally correlated with impaired spatial working memory. A HN derivative has been reported to improve cognitive impairments in different genetic mouse models of AD (Niikura et al., 2011; Zhang et al., 2012) as well as blunt learning and memory decline induced by Aβ peptides (Tajima et al., 2005). Also, HN ameliorates spatial working memory deficits induced by cholinergic antagonism- and GABA agonist-induced amnesia in mice (Mamiya and Ukai, 2001; Krejcova et al., 2004; Tajima et al., 2005). It has been recently reported that HN treatment improves cognition in aged mice. Also, there is a positive correlation between decreased HN circulating levels and accelerated cognitive aging in humans (Yen et al., 2018). Moreover, intrahippocampal injection of HN into the CA1 region is able to prevent Aβ-induced memory deficits (Chai et al., 2014). Thus, it can be hypothesized that decreased HN expression in the CA1 region of the hippocampus could be the underlying mechanism for cognitive impairment induced by OVX.

It is well recognized that structural synaptic plasticity, which involves changes in synaptic architecture and number, is an important biological basis of learning and memory (Lamprecht and LeDoux, 2004). *In vivo* treatment with HN has been shown to prevent Aβ-induced dendritic atrophy in the CA1 region of the hippocampus by promoting dendritic branching and spine density. These HN-induced effects occur concomitantly with an increase in pre- and post-synaptic proteins in this hippocampal subregion (Chai et al., 2014). To our knowledge, ours is the first *in vitro* study showing that HN has a direct effect at the synaptic level preventing glutamate-induced structural synaptic alterations in hippocampal neurons.

HN has been shown to protect cultured rat cortical neurons from NMDA-induced neurotoxicity, an effect that seems to be time- and concentration-dependent (Cui et al., 2014, 2017; Yang et al., 2018). In this study, we aimed at studying HN effects on structural synaptic plasticity in glutamate-induced dendritic atrophy and synapse alterations (Podestá et al., 2014). This approach, which consists on subjecting cultured hippocampal neurons to a brief exposure to a low glutamate concentration, proved to be mediated by NMDA receptor (Podestá et al., 2014). Our results show that HN is able to prevent glutamate-induced dendritic atrophy even at the lowest concentration studied. Interestingly, we also detected a reduction in synapse number, evidenced by decreased SYN puncta number and total SYN puncta area, which occurs simultaneously with dendritic retraction. Remarkably, HN prevented these structural synaptic alterations at concentrations that are comparable to HNr levels detected in astrocyte conditioned media by ELISA, suggesting that HN could be exerting its actions in pathophysiological conditions.

In summary, our results indicate that long-term ovarian hormone deprivation promotes structural changes in hippocampal astrocytes, which is positively correlated with reduced HN expression in this brain area. Our results *in vitro* show that ovarian hormones positively regulate astroglial HN expression and release and that this peptide prevents glutamate-induced structural synaptic alterations of cultured hippocampal neurons. Thus, OVX-induced functional and morphological alterations in astrocytes discussed above could impair astroglial support to neuronal function and may represent an underlying mechanism for synaptic dysfunction after menopause. Our study could help find new therapeutic targets for interventions that may promote a healthier lifespan for post-menopausal women.

## Ethics Statement

Animals were kept in accordance with the National Institutes of Health Guide for the Care and Use of Laboratory Animals. Animal protocols were previously approved by the Ethics Committee of the School of Medicine, University of Buenos Aires (Res. N° 2249).

## Author Contributions

SZ and AR contributed to the conception and design of the study. SZ and MT performed the experiments. MC contributed to the design and performance of behavioral tests. SZ wrote the first draft of the manuscript. SZ, AR and AS discussed results. AR and AS revised the first draft of the manuscript. All authors contributed to the revision of the final version of the manuscript, read and approved the submitted version.

## Conflict of Interest Statement

The authors declare that the research was conducted in the absence of any commercial or financial relationships that could be construed as a potential conflict of interest. The handling Editor declared a shared affiliation, though no other collaboration, with the authors.

## Supplementary Material

FIGURE S1 Validation of the 2^−ΔΔCt^ method for quantitative polymerase chain reaction (qPCR) data quantification. The efficiency of amplification of **(A)** the target gene (HNr) and **(B)** housekeeping control (HPRT) was examined using real-time PCR and SYBR Green detection. Using reverse transcriptase, cDNA was synthesized from 1 μg total RNA isolated from sham-operated (SHAM) and ovariectomized (OVX) rat hippocampi. Serial dilutions of cDNA were amplified by real-time PCR using gene-specific primers. The most concentrated sample contained 200 ng of cDNA. **(C)** The ΔCT (Ct_HNr_-Ct_HPRT_) was calculated for each cDNA dilution. The data were fit using least-squares linear regression analysis (*n* = 2–3). **(D)** Validation of HPRT as housekeeping gene. The expression of HPRT RNA was evaluated in hippocampi from SHAM and OVX rats by RT-qPCR. Each column represents the mean ± SEM of the concentration of HPRT RNA expressed as fold-changes relative to SHAM group (AU; *n* = 3 animals/group); *p* = 0.0802, Student’s *t*-test.

FIGURE S2 Negative controls for immunohistochemistry assays. Coronal sections from the hippocampus of SHAM or OVX animals were processed for double immunohistochemistry for HNr and GFAP. **(A)** Representative microphotographs from CA1 stratum oriens show the lack of signal in the 488 (green) and 594 (red) channels in sections incubated only with secondary antibodies (double negative control). **(B)** Representative microphotographs from CA1 stratum oriens show the expression of GFAP (green) or HNr (red) together with the lack of signal in the 594 (red) or 488 (green) channels respectively in sections incubated in the presence of each primary antibody as indicated in the figure and both secondary antibodies (single negative control). Nuclear staining is shown in blue (DAPI). Scale bar = 50 μm.

FIGURE S3 Negative controls for immunocytochemistry assays. Hippocampal neurons in culture (DIV 13) were immunostained for double immunohistochemistry for MAP-2 and SYN. **(A)** Representative microphotographs show the lack of signal in the 488 (green) and 590 (red) channels in neurons incubated only with secondary antibodies (double negative control). **(B)** Representative microphotographs show the expression of SYN (red) or MAP-2 (green) together with the lack of signal in the 488 (green) or 590 (red) channels respectively in neurons incubated in the presence of each primary antibody as indicated in the figure and both secondary antibodies (single negative control). Nuclear staining is shown in blue (DAPI). Scale bar = 50 μm.

## Abbreviations

CNS: Central nervous system
GFAP: glial fibrillary acidic protein
HN: humanin
HNr: rattin
AD: Alzheimer’s disease
ORF: open reading frame
rRNA: ribosomal RNA
NMDA: *N*-methyl-D-aspartate
CNTFR-α: ciliary neurotrophic factor α
DMEM: Dulbecco’s Modified Eagle Medium
FCS: fetal calf serum
OVX: ovariectomized
HPRT: hypoxanthine-guanine phosphoribosyltransferase
FITC: fluorescein isothiocyanate
DAPI: 4′,6 diamidino-2-phenilindoledihydrocloride
E: 17β-estradiol
P: progesterone
MTT: 3-(4, 5-dimethylthiazol-2-yl)-2, 5-diphenyltetrazolium bromide
DIV: day *in vitro*
GH: growth hormone
IGF-1: insulin-like growth factor-1
TFAM: mitochondrial transcription factor A
mtDNA: mitochondrial DNA
DG: dentate gyrus
GABA: γ-aminobutyric acid.
