# Supplementary figures and images for: Humanin, a Mitochondrial-Derived Peptide Released by Astrocytes, Prevents Synapse Loss in Hippocampal Neurons
Source: Front Aging Neurosci. 2019 May 31;11:123. doi: 10.3389/fnagi.2019.00123 (PMC6555273; doi:10.3389/fnagi.2019.00123)

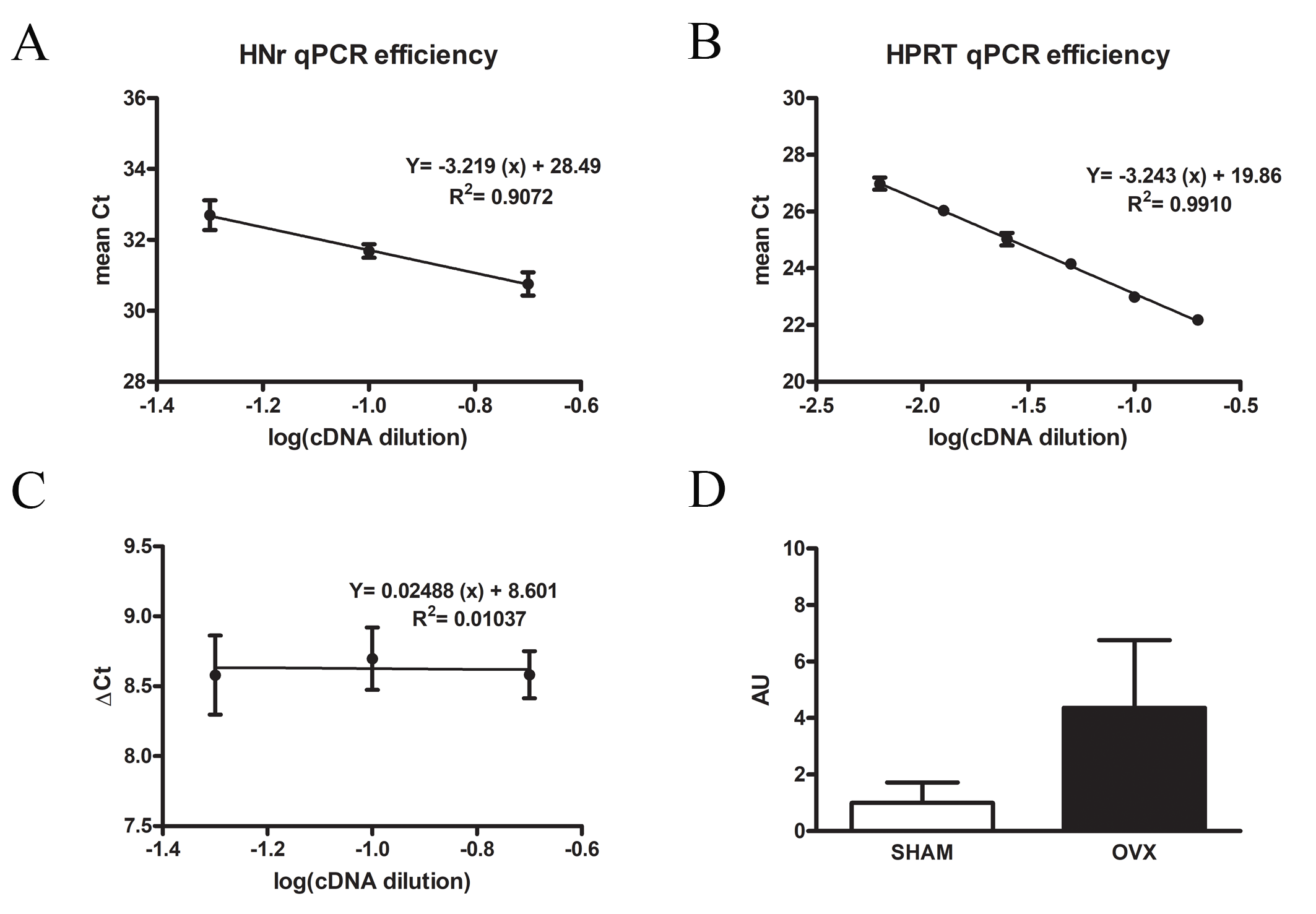

Supplement: FIGURE S1 — Validation of the 2−ΔΔCt method for quantitative polymerase chain reaction (qPCR) data quantification. The efficiency of amplification of (A) the target gene (HNr) and (B) housekeeping control (HPRT) was examined using real-time PCR and SYBR Green detection. Using reverse transcriptase, cDNA was synthesized from 1 μg total RNA isolated from sham-operated (SHAM) and ovariectomized (OVX) rat hippocampi. Serial dilutions of cDNA were amplified by real-time PCR using gene-specific primers. The most concentrated sample contained 200 ng of cDNA. (C) The ΔCT (CtHNr-CtHPRT) was calculated for each cDNA dilution. The data were fit using least-squares linear regression analysis (n = 2–3). (D) Validation of HPRT as housekeeping gene. The expression of HPRT RNA was evaluated in hippocampi from SHAM and OVX rats by RT-qPCR. Each column represents the mean ± SEM of the concentration of HPRT RNA expressed as fold-changes relative to SHAM group (AU; n = 3 animals/group); p = 0.0802, Student’s t-test. [file Image_1.TIF]

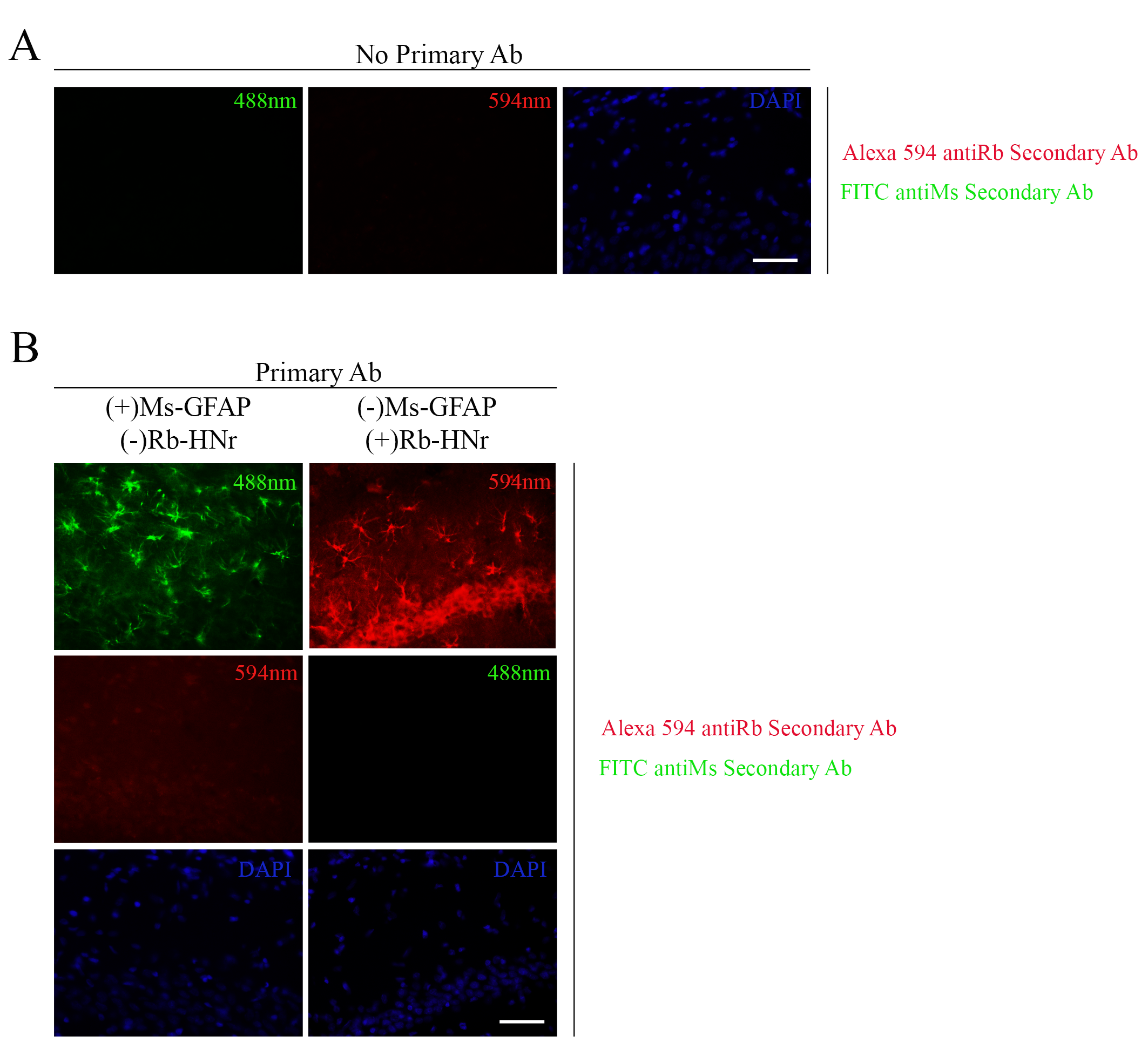

Supplement: FIGURE S2 — Negative controls for immunohistochemistry assays. Coronal sections from the hippocampus of SHAM or OVX animals were processed for double immunohistochemistry for HNr and GFAP. (A) Representative microphotographs from CA1 stratum oriens show the lack of signal in the 488 (green) and 594 (red) channels in sections incubated only with secondary antibodies (double negative control). (B) Representative microphotographs from CA1 stratum oriens show the expression of GFAP (green) or HNr (red) together with the lack of signal in the 594 (red) or 488 (green) channels respectively in sections incubated in the presence of each primary antibody as indicated in the figure and both secondary antibodies (single negative control). Nuclear staining is shown in blue (DAPI). Scale bar = 50 μm. [file Image_2.TIF]

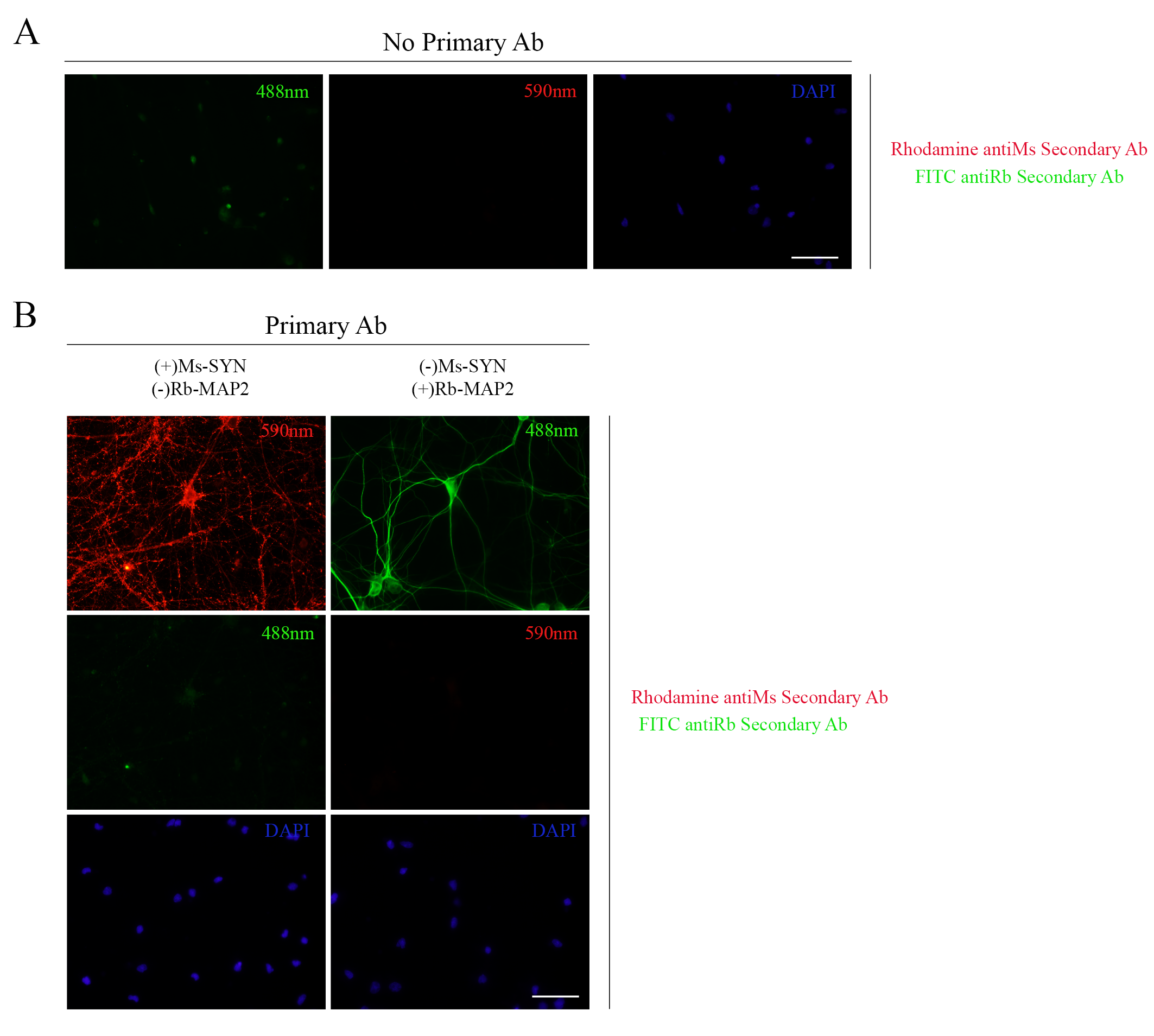

Supplement: FIGURE S3 — Negative controls for immunocytochemistry assays. Hippocampal neurons in culture (DIV 13) were immunostained for double immunohistochemistry for MAP-2 and SYN. (A) Representative microphotographs show the lack of signal in the 488 (green) and 590 (red) channels in neurons incubated only with secondary antibodies (double negative control). (B) Representative microphotographs show the expression of SYN (red) or MAP-2 (green) together with the lack of signal in the 488 (green) or 590 (red) channels respectively in neurons incubated in the presence of each primary antibody as indicated in the figure and both secondary antibodies (single negative control). Nuclear staining is shown in blue (DAPI). Scale bar = 50 μm. [file Image_3.TIF]
